# Directions for Vulcanite Work

**Published:** 1862-04

**Authors:** 


### DIRECTIONS FOR VULCANITE WORK.

In the following directions we will present a brief but comprehensive summary of the best method of constructing this work. By these directions the American Hard Rubber Company's gum, or any similar gum, may be used.

Having obtained a perfect model for a base plate, take a sheet of Gutta Percha, soften it in hot water, or by holding it before a fire, the latter is preferable, then place it upon, and press it closely to, all parts of the cast, where the plate is designed to extend; cool and trim to the desired form. Then with this, obtain the articulation according to the usual method of procedure. Now, select, grind, joint up, arrange the teeth, either single or in blocks, and try them in the mouth; the teeth being fastened to the base plate, with good adhesive wax; when their position is decided upon, place the piece on the cast, then model up the piece to the desired form with the wax, cover the pins perfectly, and close all spaces between the plate and the cast, and indeed any place where it is intended the gum should occupy. Form the base of wax, in such a shape as required when the work is finished, leaving a surplus to allow for finishing. Place the model in the lower part of the flask, and fill with plaster, up to the wax, or pattern plate; the teeth should remain in their relative position with the model; to accomplish this, build up a plaster wall, upon the labial surface of the teeth, letting it extend from the crown surfaces, to the periphery of the flask, then trim the whole smooth, giving it draft, varnish and oil it, then place on the upper section of the flask, and fill with plaster, put on the cover, and press down close.

When the plaster is set, warm sufficient to soften the wax, then separate the sections of the flask gently. Then remove the plate and wax, and cut several grooves from the plate mould in the plaster, to the border of the flask, for the escape of the surplus gum. If teeth with the common long pins are used, flatten and bend them, so they will hold firmly in the rubber; if the improved pins are used this is not necessary.

If there is wax in any part that can not be removed with instruments, the piece may be placed in boiling water, when the wax will all be melted out. Now heat up the sections, to about the point of boiling water, and keep it as near this temperature as possible, while packing. Cut the gum into pieces or strips, and lay it on a piece of cloth or paper, on a hot brick, or piece of iron, that it may keep soft while packing. The gum should be so packed in, with the appropriate instruments, as to thoroughly fill all the spaces occupied by the wax. Then put the flask together, put on a strong spring, and put it in boiling water, when in a few moments it will be perfectly closed. If this does not accomplish the closure, it may be put in a strong press, and gradually closed: it is then ready for the heater. The method of procedure now depends somewhat upon the kind of heater used, but some general directions may be given for all. The heater should always be absolutely steam tight. The piece should be placed in the receiver, with a little water; it is not necessary that the piece be covered with water if no steam escapes. The heat should be run up to 330° and retained at that for 40 minutes, then let the steam escape, take out the flask, cool it, separate carefully and ascertain if the rubber is sufficiently vulcanized, if it is, remove the piece from the flask, when it is ready for finishing; if it is not, just close the sections again without disturbing or breaking up the plaster, place it in the heater, and experience alone will direct the length of time to finish the process. The piece being vulcanized it is ready for finishing.

And for this purpose first use a half round file or rasp, where it can be used upon the piece; then with scrapers of the proper forms cut away the material to the required shape and thickness. Smooth the surface with emery or sand paper, then with pulverized pumice stone and water with a soft cork on a lathe cut away the scratches; then polish same as metal plates.

There are many particulars of minutiæ for special cases that are not given here; but for general use, we think the above will embrace all general principles.

We have from time to time given in the pages of the Register directions in detail for special cases, as they come up, and design to do so in future. T.

